# Anatomical and Biological Considerations to Determine Resectability in Pancreatic Cancer

**DOI:** 10.3390/cancers16030489

**Published:** 2024-01-23

**Authors:** Ingmar F. Rompen, Joseph R. Habib, Christopher L. Wolfgang, Ammar A. Javed

**Affiliations:** 1 Department of Surgery, The NYU Grossman School of Medicine and NYU Langone Health, New York, NY 10016, USA; 2 Department of General, Visceral, and Transplantation Surgery, Heidelberg University Hospital, 69120 Heidelberg, Germany

**Keywords:** pancreatic neoplasms, pancreatic ductal adenocarcinoma, tumor biology, biomarkers, CA19-9, resectability, biological staging

## Abstract

**Simple Summary:**

Surgical candidacy in the treatment of pancreatic cancer is complex. There are multiple factors influencing if a patient is a good candidate for surgery with or without preceding treatment with chemotherapy. This review summarizes the importance of both anatomical (tumor size and its relation to important blood vessels to the liver and bowel) and biological (aggressiveness of the tumor) considerations.

**Abstract:**

Pancreatic ductal adenocarcinoma (PDAC) remains associated with poor outcomes with a 5-year survival of 12% across all stages of the disease. These poor outcomes are driven by a delay in diagnosis and an early propensity for systemic dissemination of the disease. Recently, aggressive surgical approaches involving complex vascular resections and reconstructions have become more common, thus allowing more locally advanced tumors to be resected. Unfortunately, however, even after the completion of surgery and systemic therapy, approximately 40% of patients experience early recurrence of disease. To determine resectability, many institutions utilize anatomical staging systems based on the presence and extent of vascular involvement of major abdominal vessels around the pancreas. However, these classification systems are based on anatomical considerations only and do not factor in the burden of systemic disease. By integrating the biological criteria, we possibly could avoid futile resections often associated with significant morbidity. Especially patients with anatomically resectable disease who have a heavy burden of radiologically undetected systemic disease most likely do not derive a survival benefit from resection. On the contrary, we could offer complex resections to those who have locally advanced or oligometastatic disease but have favorable systemic biology and are most likely to benefit from resection. This review summarizes the current literature on defining anatomical and biological resectability in patients with pancreatic cancer.

## 1. Introduction

Pancreatic cancer is associated with a devastating five-year overall survival of 12% across all stages of the disease [1,2]. It is the third leading cause of cancer-related death with a rising incidence [2]. Due to the asymptomatic nature of the disease, only approximately 20% of patients are found to have resectable disease at the time of diagnosis [2,3]. Additionally, poor outcomes are driven by an early propensity for the systemic spread of the disease. This is evident by the fact that a significant proportion of patients undergoing resection will experience systemic recurrence of the disease [4,5,6]. Effective systemic control of the disease remains one of the strongest limitations to achieving cure and long-term survival.

Surgery remains the only curative therapy, with a modest survival benefit having been reported with the adjunct of multiagent system therapies. Historically, resectability has been based on anatomical considerations. The National Comprehensive Cancer Network (NCCN) guidelines define localized pancreatic cancer based on the anatomical relation between the tumor and surrounding vasculature into resectable, borderline resectable (BRPC), and locally advanced pancreatic cancer (LAPC) [7]. Advances in surgical technique and chemotherapy have increased surgical candidacy in patients with locally advanced disease, which accounts for approximately 30–40% of PDAC [8,9,10]. At specialized centers, up to 35–60% of LAPC patients can now undergo resection [11,12]. While surgical resection can provide cure and long-term survival in some patients, early recurrence with a progression of the disease is routinely seen in clinical practice [4]. In the latter cases, undetected microscopic residual disease and aggressive cancer biology may lead to early clinical recurrence and death, rendering a morbid local resection futile. Thus, considering the tumor biology to determine surgical candidacy is becoming increasingly important [13,14]. Institutions such as the international consensus meetings and the MD Anderson Cancer Center have realized the importance of tumor biology and come up with alternative criteria for resectability that factor in both anatomic and biological parameters [15,16].

Despite these considerations, significant variability exists in adherence to these guidelines across centers. Some centers advocate upfront resection to avoid progression of the disease and worsening of the patient’s condition during the neoadjuvant window while others prefer a neoadjuvant approach. The latter has the benefit of early treatment of undetected micrometastatic disease and the selection of surgical candidates by avoiding futile surgery in those who progress on systemic therapy and ensures that all patients receive systemic therapy. The variability in outcomes of patients regardless of their local stage suggests a role for tumor heterogeneity and tumor biology in driving these outcomes. Therefore, a precision approach is warranted where it is not only the anatomical staging of the disease but also a biological assessment that should determine resectability in these patients. In doing so, we would perform resections in patients who would derive survival benefit while avoiding potentially morbid operations in those with a heavy burden of systemic disease. This review aims to summarize and discuss the evidence on the anatomical and biological considerations of resectability for the management of pancreatic cancer.

## 2. Diagnostic Workup for Pancreatic Cancer 

When assessing resectability, an adequate diagnostic workup is pivotal. The gold standard for pancreatic cancer diagnosis is a pancreas protocol computed tomography (PPCT) [7,17], which can help in assessing both the locoregional extent and the metastatic spread of disease to the liver, peritoneum, and lungs [18]. It is also highly sensitive and specific for vascular involvement with venous contact or infiltration presenting with obstruction or thrombosis of the vein, stenosis or irregularity of the vessel caliber, or a teardrop sign [19]. Similarly, arterial involvement of the superior mesenteric artery (SMA), celiac trunk (CA), or the common hepatic artery (CHA) can be appreciated on PPCT and is relevant to resectability and prognosis [19]. Furthermore, PPCT can help to assess the aberrant anatomy of hepatic arteries and stenosis of the celiac trunk, which are important for surgical planning. Even after neoadjuvant treatment, the CT scan can reliably distinguish between vascular infiltration and vascular contact through the “Halo” and “String” signs [20]. The “Halo” sign describes tumor infiltration of the lymphatic and neural tissue surrounding the SMA and, therefore, arterial encasement but without true infiltration [20]. This allows for the surgical planning of periadventitial divestment, thus skeletonizing the involved artery [21]. The “String” sign on the other hand suggests true arterial invasion, and therefore, surgical planning mandatorily should involve strategies for arterial resection and reconstruction [20]. The artery-first approach allows further exploration and identification of SMA involvement before the point of no return is reached [21,22,23]. Depending on the site of predicted involvement, different approaches can be chosen [24]. However, poor performance of the CT is reported for the detection of small metastases and nodal involvement with a diagnostic accuracy of 38–77% and a sensitivity of 14–24% [19]. Both CT and magnet resonance imaging (MRI) have similar sensitivity and specificity. Due to wider availability and the greater consistency of images within CT scans, MRI is typically used in patients with contraindications for CT [25]. Currently, FDG or FAPI PET/CT is not routinely used for pancreatic cancer workup [19]. An additional value is proposed for detecting lymph node involvement and metastatic disease [25]. Additionally, cinematic rendering has shown promise in the vascular mapping and identification of occult metastases in pancreatic cancer [26]. Following an image-based diagnosis of the disease, an endoscopic ultrasound (EUS) with a fine-needle aspiration (FNA) or brush cytology is used for histopathological confirmation of the disease, which is important for the initiation of neoadjuvant/induction therapy [19,27].

Recent studies have evaluated the role of laparoscopic staging before neoadjuvant treatment in LAPC for better assessment of occult metastases or before surgical exploration to assess resectability [28]. When performed in the pretreatment setting, occult metastases are detected in around 10–20% of patients with resectable or borderline resectable and up to 30% in locally advanced PDACs [29,30]. Additionally, intraoperative ultrasound can be used to assess the tumor location and vascular involvement during exploration [31]. The NCCN guidelines recommend a staging laparoscopy directly before a planned resection through laparotomy in all patients with a high suspicion of peritoneal or hepatic metastases or questionable resectability [7]. Thus, a staging laparoscopy should be considered in patients with CA19-9 > 150 U/mL, low-volume ascites, borderline resectable tumor, size > 3 cm, or suspicious lymphadenopathy.

## 3. Anatomical Assessment of Resectability and Implications for Treatment

Once the diagnosis of PDAC is established, tumor staging is performed using the American Joint Committee on Cancer (AJCC) TNM classification [32]. For localized PDACs, TNM staging has limited value for the assessment of resectability [32]. Therefore, the AHPBA [33], Alliance [34], NCCN [7], and the MD Anderson Cancer Center [35] have developed anatomical criteria that are primarily based on the extent of involvement of the surrounding major vessels (Table 1). Radiographically localized disease is divided into resectable, borderline resectable, and locally advanced disease. In brief, resectable tumors are those that can be resected without any additional resections and reconstructions of the vessels. Borderline resectable tumors often require additional resection and reconstruction of adjacent vessels or en bloc resection of infiltrated organs and are at a higher risk of margin-positive resections [13]. In locally advanced tumors, upfront resection is extremely challenging due to the extent of vascular involvement [36]. Major differences within the classifications are that the AHPBA classification defines tumors with contact to the SMV/portal vein as borderline resectable whereas, for all other relevant classifications, encasement contact <180° is considered the resectable stage. These anatomical criteria have a prognostic value and have implications on the treatment sequence [4,36].

For resectable tumors, upfront resection with adjuvant chemotherapy, is currently preferred [36]. However, it has been shown that approximately 40% of patients do not receive adjuvant therapy due to postoperative complications or early progression of the disease [37]. Due to a substantial number of patients being diagnosed with recurrence despite successful resection, there is an increasing belief that pancreatic cancer is a systemic disease even when diagnosed at an early stage [5]. Therefore, there may be a theoretical benefit for patients receiving neoadjuvant therapy in order to control disseminated disease early on [38]. On the contrary, others argue that there is a risk of progression of the disease during the neoadjuvant window that would render these patients unresectable or lead to a worsening of their physical condition during chemotherapy, preventing the patient from undergoing a possibly curative resection [36]. To date, there is no survival benefit shown within subgroup analyses for resectable PDAC in randomized trials comparing neoadjuvant treatment versus upfront surgery such as the PREOPANC1 trial (HR 0.79, 95%CI: 0.54–1.16, *p* = 0.23) [39]. The PRODIGE48/PANACHE-01 and NEONAX phase II trials showed the feasibility of perioperative cytotoxic treatment with FOLFIRINOX or gemcitabine plus nab-paclitaxel, respectively [40,41], but the preliminary results of the NORPANC trial showed opposite trends with worse survival rates despite an improvement in R0 resection rates after neoadjuvant treatment [42]. Further ongoing studies including the ALLIANCE (NCT 04340141) and PREOPANC-3 (NL75539.078.20) will add to the growing evidence for the treatment sequence in resectable PDACs. 

For borderline resectable pancreatic cancers, there is a higher risk of a margin-positive resection when using a surgery-first approach [39]. It is reported that, of all patients started on neoadjuvant therapy, only approximately 65% will undergo resection, with a majority of the rest having progression of the disease [13]. Overall, the ESPAC-5 and PREOPANC trials showed a survival benefit with neoadjuvant treatment in borderline resectable patients [39,43]. In the PREOPANC-I study, a median survival benefit of 4.4 months (17.6 months versus 13.2 months, HR 0.62, 95%CI: 0.40–0.95) and an improved R0 resection rate (72% vs. 43%, *p* < 0.001) were observed in the intention-to-treat analysis comparing gemcitabine plus radiotherapy versus upfront surgery [39]. The survival benefit was present despite a lower resection rate, which was 61% in the neoadjuvant group and 72% in the upfront surgery group. Similar findings were seen within the ESPAC-5 trial, with chemotherapy outperforming chemoradiotherapy in most outcomes except R0 resection rates [43].

In locally advanced pancreatic cancers, induction therapy can potentially result in a downstaging of the disease and allowing resection in approximately 35–45% of the patients [44,45]. With 8–12 cycles of mFOLFIRINOX treatment at high-volume centers with experience in vascular resections, the resection rates can be up to 60% [11]. As shown by Hackert et al., resection leads to a significant improvement in survival (15.3 months after resection vs. 8.5 months after exploration alone, *p* < 0.001) [11]. Arguably, resection may have been performed more in patients with better response to therapy or smaller tumors; however, this indicates that, with appropriate patient selection, improved survival can be achieved. Favorable survival outcomes were also concluded in a multicenter study with propensity score matching (3-year OS of 31% in the resected group versus 11% in the non-resected group) [46] and in a study investigating patients with a favorable tumor biology (>40 months versus 21.4 months, *p* < 0.001) [47]. Further trials are awaited to determine the optimal length of induction treatment and to investigate the role of vascular surgery. The value of radiotherapy in pancreatic cancer is highly disputed; no evidence supports a survival benefit while it is anecdotally associated with more challenging resections due to postradiation tissue changes [36].

With an improvement in systemic control via the introduction of multiagent systemic therapies, a growing interest in resecting patients with oligometastatic disease (limited metastatic disease confined to a single distant organ) has been observed. Resection of oligometastatic cancer may be beneficial in a highly selected group of patients after induction therapy [48]. Multiple retrospective studies have reported a survival benefit after surgical resection versus no resection [49,50]. Nagai et al. from the Johns Hopkins University showed the feasibility of resection for oligometastatic PDAC to the liver with 38.1 months median survival after induction chemotherapy [49]. Furthermore, the series from the Heidelberg University Hospital showed a 5-year survival of 8.1% [50]. This suggests that a small cohort of patients with oligometastatic disease can achieve long-term survival and, hence, are optimal surgical candidates. As of now, the single-arm prospective HOLIPANC study is enrolling patients with oligometastatic liver PDAC to receive induction therapy followed by surgical exploration and resection when feasible [51]. Additionally, the randomized trial METAPANC (AIO-PAK-0219) is currently in the phase of patients acquisition for resection after induction therapy with a minimum of eight cycles of FOLFIRINOX versus continued FOLFIRINOX treatment.

Many of the aforementioned studies focus on the liver as the metastatic site; however, isolated lung oligometastatic pancreatic cancer is associated with a better prognosis than that with liver or peritoneal involvement [4,52,53]. The limited evidence that is available shows improved outcomes for primary surgery and for metastasectomy in lung-only synchronous or metachronous pancreatic cancer [52,53]. Stuart et al. reported a survival after recurrence of 30.8 months in the subgroup undergoing pulmonary metastasectomy versus 18.6 months in patients who were managed non-surgically [54]. Furthermore, an impressive median overall survival of 68.9 months after surgical treatment for lung-only recurrence was observed by Groot et al. [55]. Further studies are required to dissect the tumor biology in this cohort to identify biologically distinct subtypes. In doing so, in the future, we will be able to define cohorts that are most likely to benefit from surgical resection even in the setting of oligometastatic disease.

## 4. Biological Assessment of Resectability

**Figure 1:**
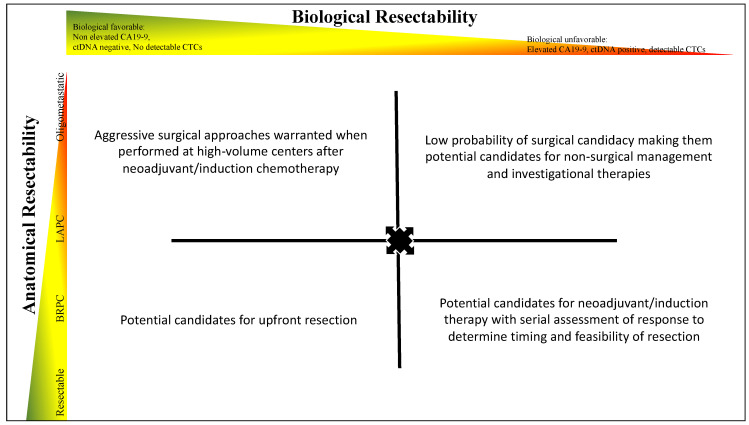
**Determination of anatomical and biological resectability.** Considerations for anatomical and biological resectability of disease. Assessment of disease is a dynamic process that involves the evaluation of anatomical and biological characteristics for each patient. For anatomical resectability, the disease can vary from resectable to oligometastatic disease, while biological resectability can be determined using biomarkers. Currently CA19-9 is used clinically, and in the future, additional biomarkers such as ctDNA, CTCs, and circulating proteins can be incorporated into this approach. At diagnosis, a patient can be categorized into one of the four quadrants on the figure, and management decisions be made accordingly. However, tumor biology is dynamic, and a serial assessment of tumor biology can lead to changes in the therapeutic approach as indicated by the arrows. For example, a patient with locally advanced pancreatic cancer and favorable tumor biology at the time of diagnosis (left upper quadrant) could progress to biologically unresectable disease during induction chemotherapy (right upper quadrant) and should, therefore, not be an appropriate surgical candidate. On the other hand, if the same patient were to remain biological resectable after induction therapy, surgical resection could be warranted even with anatomically challenging disease. Contrastingly, a patient with anatomically resectable disease that is biologically unresectable at diagnosis (right lower quadrant) could be offered neoadjuvant therapy as opposed to upfront surgery.

The anatomical considerations described earlier are vital for surgical planning and predicting local resectability. However, the systemically aggressive nature of PDAC poses a biological challenge, and multiple considerations need to be made (Figure 1). Despite substantial improvements in surgical technique and perioperative care, patient selection, therefore, should consider the tumor biology and conditional factors [56,57]. Therefore, the IAP, the MD Anderson Cancer Center and JSHBPS have published several statements regarding biological considerations for resectability [15,16,58]. In the IAP international consensus statement, the ECOG score as a surrogate conditional factor for poor tolerance to therapy was integrated as well as evidence of lymph node metastases and a cutoff value for CA19-9 (500 µ/mL) as variables for upstaging anatomical resectable disease to borderline resectable pancreatic cancer [15]. The evidence for these considerations as well as future perspectives will be discussed subsequently.

Lymph node involvement does not alter local resectability in common guidelines but is represented in the biological considerations of the IAP international consensus statement [15,16,58]. In pathological evaluations, only approximately one out of three patients have no lymph node involvement [59]. A lymph node ratio > 0.2 is a strong predictor of systemic recurrence and, therefore, represents an unfavorable tumor biology [5,59]. Furthermore, there is a strong correlation to beneficial survival effects for adjuvant chemotherapy in node-positive PDAC patients [60]. Van Roessel et al. also showed that, after neoadjuvant therapy, only patients with pathologically node-positive disease benefit from further adjuvant treatment [61]. Therefore, lymph node positivity is a strong surrogate marker for systemic disease and unfavorable tumor biology. However, given that this information becomes available after resection, it has limited utility in determining resectability. As imaging modalities improve and the preoperative prediction of nodal disease becomes accurate, this could be integrated into the biological assessment of resectability. 

Carbohydrate antigen 19-9 (CA19-9) is the most frequently used biomarker for the assessment of disease in pancreatic cancer. Its sensitivity is, however, limited by approximately 15–20% of the patients being non-producers due to having a Lewis antigen Alpha and Beta negative blood group [62]. Furthermore, often, coexistent biliary obstruction can elevate levels [28]. Nevertheless, elevated CA19-9 levels can be used as a surrogate marker for tumor burden and activity [62]. Obtaining a baseline CA19-9 can be used to predict long-term survival and correlates with R0 resections [62]. However, the most important function of CA19-9 is as a biomarker for monitoring treatment response and recurrence.

A baseline CA19-9 cutoff of 500 µ/mL was suggested by the IAP for upstaging resectable to borderline resectable PDAC [15,16]. A retrospective study of upfront-treated patients conducted by Kato et al. showed a worse prognosis for the borderline resectable stage due to biological criteria compared to resectable stages [63]. However, the prognosis was still significantly better than that for non-biological borderline tumors (NCCN borderline or resectable with ECOG > 2) [63]. The CA19-9 cutoff of 500 was not significant as a prognostic factor, but >1000 µ/mL was (OR 2.03, 95%CI: 1.45–2.84) [63]. In the PREOPANC study, there was no difference in treatment effect for patients below or above 500 µ/mL [39]. Again, validated in a combined study of two RCTs, patients with a CA19-9 > 500 µ/mL did not benefit, but patients with a CA19-9 below that threshold did actually have significant benefit from neoadjuvant treatment [64]. Since CA19-9 is a surrogate marker for tumor activity, high CA19-9 is expected in those with systemic disease [62]. However, a recent meta-analysis of recurrence patterns after neoadjuvant therapy suggests that the main benefit of neoadjuvant therapy is local control and, therefore, fewer local recurrences [65]. According to that analysis, there was no reduction in recurrences at distant sites, thus questioning the role of neoadjuvant therapy in systemic control [65]. As many studies are currently being conducted on neoadjuvant chemotherapy in resectable PDAC, further evidence on the value of CA19-9 in resectable stages is expected.

While the optimal cutoff value for upstaging resectable to borderline resectable and, thus, for having an expected benefit from neoadjuvant treatment, has still to be defined, pretreatment CA19-9 levels do not predict resectability in borderline resectable and locally advanced PDAC [66,67]. However, in a retrospective study conducted by Heger et al., a low ratio of pretreatment/posttreatment as well as lower posttreatment CA19-9 levels predicted resectability in borderline and locally advanced PDAC [67]. A posttreatment level of <91.8 U/mL predicted resectability with a 75% sensitivity and 77% specificity. A cutoff of 0.4 from posttreatment divided by pretreatment CA19-9 levels yielded a positive predictive value for resectability of 83% in patients treated with FOLFIRINOX [67]. R0 resections were achieved in 36.4% of the resected cohort, which also included oligo-metastasized staged patients. Interestingly, patients above the cutoffs did not benefit from resection versus exploration in terms of overall survival [67]. Hartlab et al. showed similar results in the NEOLAP trial; however, they stated an optimal cutoff at posttreatment level below 50 U/mL for predicting survival and <61 U/mL for R0 resection rate [66]. These results were further validated in a study from the Massachusetts General Hospital with the normalization of CA19-9 being associated with resectability [68]. Furthermore, a prospective study conducted by van Veldhuisen et al. showed a relevant benefit for adding >30% CA19-9 response to the RECIST criteria [69].

**Figure 2:**
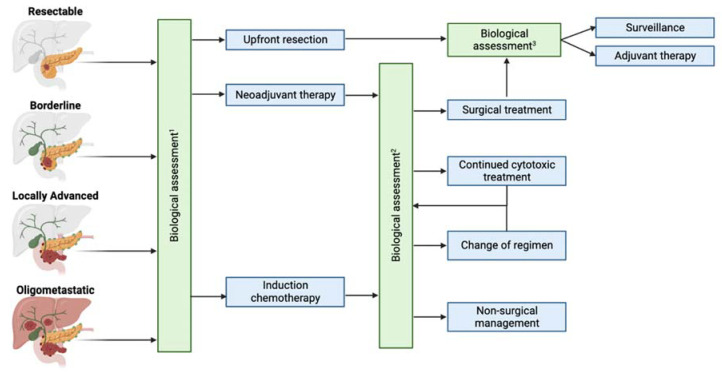
**Serial Assessment of Tumor Biology:** Serial assessment of anatomical and biological resectability can aid in the appropriate treatment for pancreatic cancer patients. (1) Determining the need for neoadjuvant therapy (anatomically resectable) and the mode of neoadjuvant/induction therapy (all anatomic stages). (2) Serial assessment of tumor response to determine surgical candidacy and the timing of resection. Local progression of disease can indicate the need for a change of regimen while systemic progression indicates the need for non-surgical management with investigational therapies or best supportive care. (3) Determining the need and mode of adjuvant treatment. Figure designed with Bio Render.

In summary, for a patient with a resectable PDAC, the value of pretreatment CA19-9 and its implications on treatment sequence have yet to be defined. In patients with borderline resectable, locally advanced, and oligometastatic cancer, the pretreatment level of CA19-9 does not predict resectability, but posttreatment level and changes between pre-and posttreatment values do. Therefore, the phase of neoadjuvant treatment provides a window of opportunity to assess tumor biology and allows the tumor to declare its aggressiveness. If a poor response is observed, the chemotherapy agents can be switched [70]. However, after exhausting all available therapies and if a poor response with a high propensity of systemic disease is observed, it could potentially indicate a biologically aggressive tumor, questioning the benefit from further cytotoxic or surgical treatments (Figure 2). Further studies are needed to define optimal cutoffs for CA19-9, such that they are standardized and broadly applicable in clinical practice. 

Carcinoembryonic antigen (CEA) is another biomarker that is used as an adjunct to CA19-9 in the clinical setting. An elevated CEA shows associations with advanced tumor stages and poor prognosis [71]. However, for the assessment of resectability, CEA shows poor performance [72]. CEA is furthermore not elevated in approximately 50–60% of PDAC patients [73,74]. Therefore, its current value for the assessment of PDAC is limited, and the need for an alternative biomarker is urgent [73]. Other potential candidate biomarkers that could help assess the tumor biology and systemic burden of the disease have been identified and are being studied. The most reported biomarkers include TIMP-1, DUPAN-2, serum-MUC5AC, CA125, and CA242 [75,76]. While CA19-9 still outperforms the novel biomarkers, biomarker panels including or excluding CA19-9 have shown improvements in diagnostic accuracy [76]. Elevated levels of DUPAN-2 (>200 U/mL) have also shown to be of prognostic value in CA19-9 non-producers [76]. DUPAN-2 is a precursor of CA19-9 and is not further metabolized in Lewis negative blood groups and, therefore, serves as a potential substitute in CA19-9 non-producers [77]. Furthermore, CEA and CA125 are associated with tumor burden and therapeutic response in CA19-9 non-producers, making them potential monitoring biomarkers [78,79]. Inflammation, as a surrogate for tumor dynamics and immunologic antitumor response, has been studied. High neutrophil/lymphocyte ratio (NLR), platelet/lymphocyte ratio (PLR), and elevated c-reactive protein (CRP) have been found to be poor prognostic factors [80,81]. Interestingly, pre-operative NLR and PLR was inversely correlated with R0 resections in a retrospective study by Recio-Boiles et al. [82]. However, these predictive values and their correlations with resectability have to be interpreted with caution. First, there is no accepted cutoff, and there is a high variability within published studies [83]. Second, there is a high possibility of publication bias as many negative results are not published [83]. In summary, the literature in predicting resectability and prognosis with alternative serum protein biomarkers or cell ratios is sparse or not convincing to date.

Through tumor biopsies, additional information can be drawn about the primary tumor, and tumor grading and molecular assessment can be performed. For example, liver recurrence is associated with poor tumor differentiation and often occurs early after surgery [18]. However, even with the recently improved understanding of the mutational landscape, convincing targetable mutations have not yet been identified. PDACs usually arises from pancreatic intraepithelial neoplasia (PanIN) precursor lesions, while some arise from intraductal papillary mucinous neoplasms (IPMNs). In PanIN-derived pancreatic cancer, usually KRAS mutation (87%) is present with later mutations involving TP53 (62%), CDKN2A (16%), and SMAD4 (16%) [84,85]. SMAD4 mutations, loss of function of the CDKN2A tumor suppressor gene, and FGFR2 gene fusions are associated with a poor outcome after standard chemotherapeutic treatment in PDACs [86,87]. Bailey et al. described four different subtypes that differ in their evolution and aggressivity [88]. According to their mutational and expression profiles, PDACs can be subclassified into squamous, ADEX, pancreatic progenitor, and immunogenic subtypes [88]. One stand out for the prognosis is the epithelial subtype, which has considerable overlap with the quasi-mesenchymal subtype described by Collissons et al. [89] The squamous subtype is characterized by gene networks that are involved in inflammation, hypoxia response, metabolic reprogramming, TGF-B signaling, MYC pathway activation, autophagy, and upregulated expression of TP63 [88]. Many of those pathways are involved in epithelial–mesenchymal transition and tumor dormancy [90]. This leads to more adaptive tumor cells and results in more treatment failure and poorer outcomes compared to the classical subtypes [91]. Therefore, in future, subtyping may aid in treatment decisions concerning biological resectability. 

Liquid biopsy has emerged as a promising technology that allows the non-invasive sampling of tumor fragments within the blood circulation including circulating tumor DNA (ctDNA), microRNA, tumor-derived exosomes, and circulating tumor cells (CTCs) [62]. The analysis of liquid biopsies can inform treatment decisions as it harbors specific information about the tumor biology such as specific mutations and epigenetic changes from the primary tumor or metastatic sites [62]. In the future, it can possibly overcome the drawback of tumor biopsies with limited identification of tumor heterogeneity as it represents the features of the systemic involvement of disease [92]. As a reliable marker for systemic disease, it most possibly fails to detect local-only disease, limiting its diagnostic value. Therefore, in a current meta-analysis, pooled sensitivity for ctDNA and CTC is low with 64% and 74% with a higher specificity of 92% and 83%, respectively [93]. 

Circulating tumor DNA is defined as the tumor-derived portion of cell free DNA within the circulation. Through apoptosis and necrosis within the tumor, ctDNA is released into the circulation [94]. The ctDNA-fragments are isolated, amplified, and analyzed with PCR or sequencing [92]. With an approximate mutation rate of 90%, KRAS (most polymorphisms G12D, G12V, and G12R) is the most important ctDNA marker, but panels of different mutations can also be used [92,94]. Low concentrations and mutations in premalignant lesions hamper its value as a diagnostic biomarker. However, ctDNA has shown valuable results as a diagnostic, monitoring, and prognostic biomarker in many cancer types [95,96,97]. When detected at baseline, it predicts worse progression-free and overall survival [98]. Longitudinal multigene ctDNA measurements furthermore predicted progression through increasing levels in 70% of patients with advanced PDAC in the study of Lapin et al. [98]. The lead time to radiologically determined progression was 19 days compared to 6 days in CA19-9 increase [98]. Therefore, it could possibly be used as a monitoring biomarker also in neoadjuvant treatment as ctDNA persistence was associated with treatment failure to cytotoxic agents [98]. 

In terms of predicting resectability, ctDNA, to date, has not been implemented in clinical decision making. Higher ctDNA levels are associated with vascular involvement and advanced local tumor size [99]. Furthermore, ctDNA was correlated with positive resection margins within the study of McDuff et al. [100]. In LAPC, ctDNA-positive patients had 44% R0 resections compared to 88% with ctDNA-negative findings (*n* = 29) [100]. In summary, since preoperative ctDNA positivity has shown to be a predictor of early recurrence and worse survival outcomes in resectable PDAC, its positivity could aid the selection of resectable PDAC patients who will benefit from neoadjuvant treatment compared to upfront resection [100,101].

Circulating tumor cells are tumor cells that have detached from the primary tumor or metastatic site and can be found within the blood stream. Epithelial–mesenchymal transition (EMT) can lead to cell migration, intravasation, and consequently, the presence of CTCs, which are key players in the early dissemination and metastatic seeding of pancreatic cancer cells [102,103]. The detection and isolation of CTCs can be performed by biomarker-mediated platforms such as microfluidic chips, magnetic beads, or size-sensitive microfiltration [104]. Analysis can then be performed, applying a wide range of methods including flow cytometry, immunofluorescence staining, and single-cell RNA sequencing (scRNAseq) [104]. As with ctDNA, the presence of CTCs with mesenchymal properties predicts recurrence after pancreatectomy [105,106]. This wide arrange of analytical method and the CTCs representing systemic disease of a heterogenous tumor may allow precision oncology in future. Anatomical resectable PDACs with positive transitional CTCs may benefit from neoadjuvant systemic treatment compared to upfront surgery.

## 5. Current Limitations of Assessment of Anatomical and Biological Resectability 

To date, there are certain limitations to the assessment of biological resectability that urgently need to be addressed. First, the radiographic assessment of lymph node involvement is unreliable. The enlargement of lymph nodes beyond the cutoff of 1 cm is deemed as suspicious for lymph node involvement in cancer. Since the enlargement of lymph nodes can also be due to peritumoral inflammation and, on the other hand, metastatic lymph nodes can measure below the cutoff value of 10 mm, the poor performance of CT scans in terms of diagnostic accuracy (38–77%) and sensitivity (14–24%) is seen in clinical practice [19]. In the future, more adequate lymph node examinations, for example, through biopsies or PET-CT may fill the gap between the reliability of radiological to pathological assessment for the decision on neoadjuvant treatment in resectable PDACs [19].

Second, the assessment of treatment response is challenging. Radiological response assessment is performed through a CT scan using the Response Evaluation Criteria in Solid Tumors (RECIST) [107]. Progression according to the RECIST criteria encompasses a tumor growth of at least 20% in diameter or newly detected lesions; stable disease is defined by the absence of progression or regression; and regression is defined as a shrinkage of at least 30% in three directions [107]. The percentages of patients with progression, stable disease, partial, and complete response after induction treatment with mFOLFIRINOX were 16%, 59%, 16%, and <1%, respectively [19,28]. However, radiological assessment after neoadjuvant treatment underestimates resectability [108,109]. In the interpretation of radiological findings, the assessment of regression is challenging due to fibrotic and edematous posttreatment reactions that are hard to distinguish from viable tumors, especially at the tumor borders [19]. Therefore, the radiologic and pathologic evaluations of tumor response only show moderate agreement [110]. In a case series by Ferrone et al., 92% of patients with LAPC had an R0 resection even though imaging suggested non-resectability [109]. Furthermore, there are no reliable predictors of resectability after neoadjuvant or induction treatment for LAPC and borderline resectable PDAC [111]. Therefore, surgical exploration should be performed in all fit patients without progression or secondary metastatic disease after induction therapy at a high-volume center with experience in arterial divestment and resection [28,36]. 

Third, in current clinical practice, the biological treatment response is mainly determined by measurement of the CA19-9 levels [112]. Since alternative serum protein biomarkers have failed to compensate for the drawbacks of CA19-9, this represents an unsatisfactory state. Efforts are currently being undertaken, especially with liquid biopsies, to compensate for those shortcomings. For example, Meijer et al. have shown that the downregulation of microRNA-181a-5p can be used to monitor the response to FOLFIRINOX [113]. Further data on liquid biopsies, especially in the setting of neoadjuvant therapy, are much awaited. Molecular analyses have yet shown very few targetable lesions or implications about different treatments. Possibly, there is an advantage in immunotherapy for the immunogenic subtype that has more immune-cell infiltration compared to the other types being immunogenic cold tumors [88]. Other efforts toward personalized treatment, for example, better treatment response to platinum-based chemotherapy, are currently under investigation [114]. In the future, multianalyte panels encompassing a range of variables have the potential to mitigate the limitations associated with solitary biomarkers.

## 6. Conclusions

Assessment of local resectability based on presence and extent of vascular involvement of major abdominal vessels has prognostic value. However, due to advancements in surgical technique including vascular resections, biological considerations are becoming even more important for treatment decision making in patients with pancreatic cancer. Patients who present with anatomically resectable disease and unfavorable tumor biology may experience early recurrence and therefore potentially morbid resection might have limited values in these cases. Conversely, patients with anatomically advanced tumors exhibiting favorable tumor biology have the potential to attain cure and long-term survival through surgical intervention. Therefore, if treatment decisions are solely guided by anatomic criteria, a risk of surgically overtreating biologically aggressive diseases and undertreating those with biologically favorable profiles exists. 

The assessment of tumor biology can be performed through surrogate markers indicative of advanced disease and systemic involvement such as CA19-9, ctDNA, and CTCs, but, to date, a majority of these biomarkers have performed insufficiently for predicting treatment benefit when analyzed alone. However, a combination of the aforementioned biomarkers could possibly define candidates who will benefit from resection compared to those who probably will develop early systemic recurrence and have dismal prognosis despite surgical treatment. Developing multianalyte composite tests based on these biomarkers is essential for defining optimal personalized treatment. Future guidelines combining anatomic and biologic features in the determination of resectability could optimize surgical candidacy such that patients undergoing resection derive maximum benefit from these operations. If a patient progresses anatomically or biological unresectability persists, this patient might not be an appropriate candidate for surgical resection. However, if a patient with LAPC with favorable tumor biology does not progress during induction chemotherapy, resection could be offered at specialized centers.

## Figures and Tables

**Table 1:** Different definitions of borderline resectable pancreatic cancer.

Borderline Resectable	NCCN	AHBPA	Alliance	IAP	MD Anderson
Superior mesenteric Vein/Portal Vein	Reconstructable involvement (distortion, narrowing, occlusion, thrombosis)	Reconstructable abutment, encasement or occlusion	Solid contact >180° or reconstructable occlusion	SMV/PV: tumor contact 180° or greater or bilateral narrowing/occlusion, not exceeding the inferior border of the duodenum	Short-segment occlusion with suitable vessel for reconstruction
Superior mesenteric artery	Solid tumor contact ≤180°	Abutment	Interface between tumor and vessel measuring < 180°	Tumor contact of less than 180° without showing deformity/stenosis	Abutment ≤ 180°
Common hepatic artery	Solid tumor contact without extension to the coeliac artery or hepatic artery bifurcation	Abutment or short-segment encasement	Reconstructable, short-segment interface between tumor and vessel of any degree	Tumor contact without showing tumor contact of the PHA and/or CA	Short-segment encasement/abutment
Celiac trunk	Solid tumor contact <180°	No abutment or encasement	Interface between tumor and vessel measuring <180°	Tumor contact of less than 180° without showing deformity/stenosis	Abutment ≤ 180°
Biological	-			Suspicion for distant metastasis, including CA19-9 > 500 U/mL, or regional lymph node metastasis diagnosed by biopsy or PET-CT	CT findings suspicious of metastatic disease; nodal-positive disease.
Conditional	-			ECOG performance status of 2 or more	Performance status ≥ 3 or severe preexisting medical comorbidity

Legend: Anatomic resectability as defined by tumor contact to abdominal vessels by the National Comprehensive Cancer Network (NCCN), Americas Hepato-Pancreato-Biliary Association (AHBPA), Alliance, International Association of Pancreatology (IAP), and MD Anderson Cancer Center. Abbreviations: SMV, superior mesenteric vein; PV, portal vein; PHA, proper hepatic artery; CA, common hepatic artery; ECOG, Eastern Cooperative Oncology Group.
